# A Comparison of Effects of Propofol and Isoflurane on Arterial Oxygenation Pressure, Mean Arterial Pressure and Heart Rate Variations Following One-Lung Ventilation in Thoracic Surgeries

**DOI:** 10.5812/ircmj.15809

**Published:** 2014-02-08

**Authors:** Alireza Sharifian Attar, Masoomeh Tabari, Mohammadreza Rahnamazadeh, Maryam Salehi

**Affiliations:** 1 Department of Anesthesiology, Ghaem Hospital, Faculty of Medicine, Mashhad University of Medical Sciences, Mashhad, IR Iran; 2 Department of Community Medicine, Faculty of Medicine, Mashhad University of Medical Sciences, Mashhad, IR Iran

**Keywords:** Propofol, Isoflurane, Arterial, One Lung Ventilation, Arterial Pressure, Heart Rate

## Abstract

**Background:**

Hypoxia occurs during one-lung ventilation (OLV) due to the arteriovenous shunt of unsaturated pulmonary venous blood. Hypoxic pulmonary vasoconstriction (HPV) acts as a defense mechanism against shunting. In thoracic surgery, anesthetics with minimal inhibitory effect on HPV and minimal hemodynamic changes are preferred.

**Objectives:**

The present study aimed to evaluate the effects of propofol and isoflurane on patients’ arterial oxygen pressure following one-lung ventilation during thoracic surgeries.

**Materials and Methods:**

In this randomized clinical trial study which was conducted in Iran, sixty patients with ASA (The American Society of Anesthesiologists) class I & II who were candidates for right elective thoracotomy were divided in two groups. Induction of anesthesia in the two groups was conducted using the same method, and left double-lumen endotracheal tube was inserted. In the first group propofol was used for the maintenance of anesthesia, and isoflurane for the second group. During two-lung ventilation and at minutes 5 and 10 after OLV, ABG (arterial blood gas) (for detecting the mean pressure of arterial oxygen), mean arterial pressure and heart rate were recorded.

**Results:**

Sixty patients (mean age = 4124.18 ± 18.63 years) were divided into two groups. The age and gender of the subjects were not statistically different between the two groups. In the propofol group, the arterial oxygen pressure during two-lung ventilation and at 5th and 10th minutes after OLV was 263.14 ± 136.19, 217.40 ± 133.99 and 182.34 ± 122.39; in the isoflurane group, it was reported as 206.29 ± 135.59, 164.78 ± 118.90 and 155.35 ± 109.21 mmHg, respectively. In the propofol group, mean arterial pressure during two-lung ventilation, and 5th and 10th minutes after OLV, was 84.01 ± 20.67, 88.15 ± 20.23 and 86.10 ± 19.13, respectively; regarding the isoflurane group, it was reported as 79.66 ± 17.04, 84.78 ± 20.19 and 86.50 ± 17.07 mmHg, respectively. In the propofol group, heart rate during two-lung ventilation, and 5th and 10th minutes after OLV was 92.77 ± 17.20, 94.0 ± 18.34 and 94.33 ± 21.03, respectively; In the isoflurane group, it was reported as 92.87 ± 16.96, 91.8 ± 18.75 and 91.05 ± 17.20 min, respectively. These values were statistically similar in the two study groups.

**Conclusions:**

The effects of propofol on hemodynamics and arterial oxygen pressure during one- or two-lung ventilation were not different from those of isoflurane.

## 1. Background

Hypoxemia during the one-lung ventilation (OLV) is a major concern in the management of anesthesia for thoracic surgery. Significant drop in arterial oxygen saturation (SPO_2_<90 %) during one-lung ventilation occurs in one to ten percent of population undergoing thoracic surgery in the presence of FIO_2_ = 100 % (fraction = 1.0). The pulmonary arteriovenous shunt of unsaturated blood is the main cause of hypoxemia during one-lung ventilation which is not ventilated. Hypoxic pulmonary vasoconstriction (HPV) is the most important defense mechanism against shunting (1). According to the different effects of anesthetics on the inhibition of HPV, it is crucial to use drugs with the minimal inhibitory effects on this vital mechanism (2). Therefore, it may be necessary to evaluate the effects of the two major anesthetic maintenance agents on HPV and their subsequent pressure of arterial oxygen using propofol (intravenous anesthetic) and isoflurane (inhaled anesthetic). It is obvious that the impact of this mechanism will emerge as SPO2 changes (2). Despite the extensive use of these drugs, their cardiovascular effects have not been thoroughly evaluated during OLV for thoracic surgery. Thus, it seems necessary that the effects of these drugs (propofol and isoflurane) should be examined on systemic hemodynamics (mean arterial blood pressure and heart rate).

## 2. Objectives

The present study aimed to evaluate the effects of propofol and isoflurane on patients’ arterial oxygen pressure following one-lung ventilation during thoracic surgeries.

## 3. Materials and Methods

In this randomized clinical trial 60 patients with ASA class I and II who were the candidates for right elective thoracotomy were recruited for lung resection in two groups of intervention and control. The calculation of sample size of the study, with two-tailed α error of 5 % and β error of 20 %, was done based on PaO_2_ measured during OLV under propofol anaesthesia in a published study [123.7 (54.7) mmHg] (3). Based on this measurement, thirty patients per group were required to detect a difference of 39.7 mmHg in the lowest PaO2 between two groups. The method of sampling was convenience and non-probability.

### 3.1. Eligibility

The studied population consisted of the patients referred to our subspecialty teaching Hospital, Mashhad, Iran to undergo elective thoracic surgery and OLV. Field data collection using checklist and direct observation by an operating room technician unaware of the study protocol and its objectives Inclusion criteria included age between 18 and 75 years, ASA class I and II, satisfaction of thoracic surgery with OLV complied with the conditions of the study. Exclusion criteria included liver dysfunction (AST > 40 and ALT > 40), ischemic or valvular heart disease (heart disease was examined by medical history, physical condition, ECG and echocardiography), end-stage obstructive or restrictive pulmonary disease, patients with OLV less than 30 minutes, patients who showed a ETCO_2_ > 45 with respiratory rate of 12 breaths/minutes and patients who had a pathological lesion in left lung (dependent lung) in preoperative assessment including HRCT and PFT.

### 3.2. Randomization

Randomization was simple and accomplished by using random assignment tables.

### 3.3. Endpoints

The arterial oxygen pressure were evaluated as primary endpoints: 10 minutes after initiation of two-lung ventilation and 5 and 10 minutes after the start of one-lung ventilation. Hemodynamic parameters were evaluated as secondary endpoints: average of mean arterial blood pressure and heart rate assessing 10 minutes after initiation of two-lung ventilation and 5 and 10 minutes after the start of one-lung ventilation.

### 3.4. Intervention

After taking a history, the required description of the research was given to the patients and the informed consent was obtained. After preoxygenation in all patients, anesthesia was induced with sodium thiopental (4 mg/kg), sufentanil (0.2 μg / kg) and atracurium (0.5 mg / kg). Initially left-sided double-lumen tube with MPI brands (Medicoplast GmbH factory) was placed for the patients, the proper placement was determined by auscultation and fiberoptic bronchoscopy. After a positional change to the lateral decubitus, the proper position was confirmed again by auscultation and fiberoptic bronchoscopy before the start of OLV. Ventilator settings were similar in all patients during a two - lung ventilation (TLV) and OLV and included:

Tidal volume (TV): 6 cc/kg, respiratory rate (RR = 12) (to maintain ETCO2 between 35 and 45), inspiratory/expiratory ratio was 1:2 (I/E = 1/2), the fraction of inspired oxygen (FIO_2_) = 1. During surgery, patients randomly received propofol (100 μg /kg/min) or one Mac (1.1 %) isoflurane as a maintenance drug based on their group. For all patients, BIS was maintained between 40 and 60 and the maintenance dosage adjustment was made if necessary. Fluid deficit was compensated with normal saline or Ringer's lactate using the "4-2-1" rule for every hour fasting before induction of anesthesia.

Monitoring included electrocardiography (ECG), end-tidal carbon dioxide tension (PETCO_2_), saturation of peripheral oxygen (SPO_2_), BIS and invasive arterial blood pressure (IBP) inserting radial artery catheter. In this step, ABG was performed twice: The first phase (during two-lung ventilation (TLV)): In this phase, 10 minutes after positioning in the left lateral decubitus during TLV. The second phase (during the first 10 minutes of OLV): in this phase, OLV is initiated and then ABG was performed at 5 and 10 minutes after the start of OLV. SpO_2_ was monitored continuously during this period, and the surgeon was allowed to open the chest. If the SPO_2_ was 90 % or less, ABG would be taken immediately and the two-lung ventilation (TLV) was restored. This arterial blood sample was considered as the lowest patient's SPO_2_ and more blood samples were not taken for further study. ABG evaluation was done using GEM® Premier™ 3000 (Instrumentation Laboratory, The Netherlands) and SPO_2_ and BP were measured by operating room patient monitoring system (SAZGAN model VECTRA, Tehran, Iran)

### 3.5. Statistical Analysis

Data description was done using percentage, frequency and relevant descriptive graphs and we used Chi-square test and t-test for data analysis when assessment of normality by Kolmogrov-Smirnov test showed normal distribution of the selected variable. SPSS software, version 16, was used for statistical analysis and in all statistical measurements P value less than 0.05 was considered significant.

### 3.6. Ethical Consideration

The current study protocol was approved by ethical committee of mashhad university of medical sciences (No. 910182, 2013/2/2 ) and All the patients gave their written informed consent to the procedure and they were ensured that their confidentiality will be kept on their personal information, in any circumstance, will not disclosed or given to any third party.

## 4. Results

**Figure 1.**
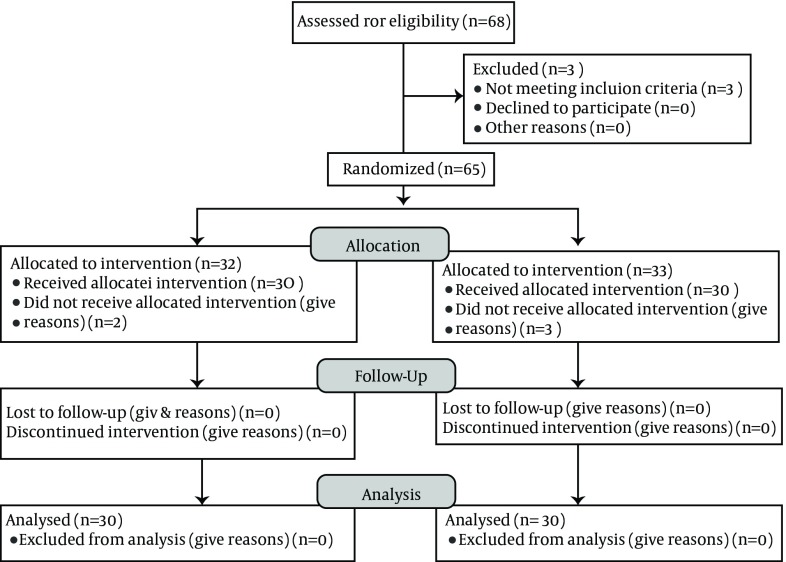
Consort Flow Diagram of the Study

**Figure 2.**
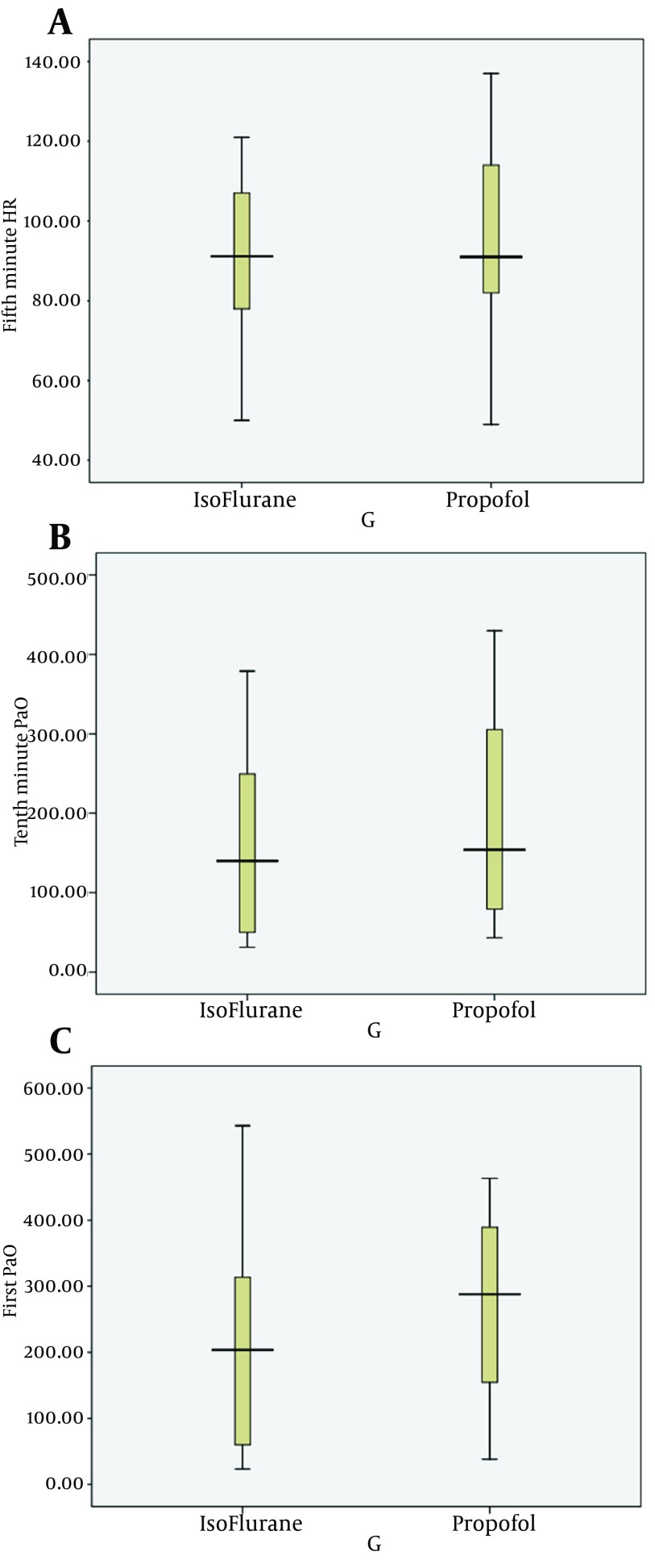
The PaO_2_ of the Subjects in Two Groups in Zero, Five and Ten Minutes After one Lung Ventilation

Sixty patients, mean age 41.24 ± 18.63 years, were enrolled in this study. There was no statistical differences (P = 0.782) in mean age between two groups (propofol group = 40.52 ± 19.20 years and isoflurane group = 41.93 ± 18.38 years). There were 23 females (38.3 %) and 37 males (61.7 %). The results showed no statistically significant difference (P = 0.426) in terms of gender distribution between two groups (in propofol group, 66.67 % males and isoflurane group, 43.33 % males). We found no differences between two groups concerning the pressure of arterial oxygen during TLV and 5 and 10 minutes after the start of OLV (Table 1 and Figure 1). There were no significant differences in mean arterial pressure and heart rate during TLV and also 5 and 10 minutes after the start of OLV in both groups (Tables 2 and 3). The diagrams of Po2 in various measured time interval is shown in Figure 2. And there was no significant difference between them in two groups of the study.

**Table 1.** Pressure of arterial oxygen in participants ^a^

	Propofol	Isoflurane	Total	P value
**TLV**	263.14 ± 136.19	206.29 ± 135.59	234.71 ± 137.75	0.111
**OLV** **− 5 min**	217.40 ± 133.99	164.78 ± 118.90	191.09 ± 128.37	0.113
**OLV – 10 min**	182.34 ± 122.39	155.35 ± 109.21	168.84 ± 115.80	0.371

^a^ Abbreviations: TLV: Two-lung ventilation; OLV: One-lung ventilation

**Table 2.** Mean Arterial Pressure in Participants ^a^

	Propofol	Isoflurane	Total	P value
**TLV**	84.01 ± 20.67	79.66 ± 17.04	81.54 ± 18.91	0.378
**OLV − 5 min**	88.15 ± 20.23	84.78 ± 20.19	86.47 ± 20.11	0.521
**OLV – 10 min**	86.10 ± 19.13	86.50 ± 17.07	86.30 ± 17.97	0.931

^a^ Abbreviations: TLV: Two-lung ventilation; OLV: One-lung ventilation

**Table 3.** Heart Rate in Participants ^a^

	Propofol	Isoflurane	Total	P value
**TLV**	92.77 ± 17.20	92.87 ± 16.96	92.82 ± 17.43	0.983
**OLV − 5 min**	94.33 ± 21.03	91.28 ± 18.75	92.81 ± 19.82	0.556
**OLV – 10 min**	94.30 ± 18.34	91.05 ± 17.20	92.67 ± 17.70	0.482

^a^ Abbreviations: TLV: Two-lung ventilation; OLV: One-lung ventilation

## 5. Discussion

Our study showed that despite the higher pressure of arterial oxygen of propofol during one-lung ventilation compared to isoflurane, there was no significant difference in both groups (pressure of arterial oxygen was 217.40 ± 133.99 at 5 minutes and 182.34 ± 122.39 at 10 minutes during one-lung ventilation in propofol group and 164.78 ± 118.90 and 155.35 ± 109.21 at 5 and 10 minutes in isuflurane group, respectively. Hemodynamic parameters including heart rate and mean arterial pressure did not show any meaningful differences during one-lung and two- lung ventilations between two groups. A clinical trial performed by J Y Wang et al. investigated the effect of isoflurane and sevoflurane on arterial oxygenation during OLV. They demonstrated that there was no difference between influence of the sevoflurane and isoflurane on arterial oxygen variations (4). In another study, Craig W. Reid and colleagues (5) compared the propofol vs. alfentanil effect in combination with isoflurane on arterial oxygenation during OLV. In addition, in 2007, this issue was evaluated by Pruszkowski (6). They compared the effect of propofol and sevoflurane on arterial oxygenation during OLV in 80 patients which demonstrated the same effect of these two maintenance drugs on pressure of arterial oxygen (PaO_2_) during OLV. The results of all of these studies were in concordance with our research. Therefore, we can conclude that PaO_2_ does not change with either propofol or inhaled anesthetic agents (such as sevoflurane, isoflurane or halothane) as a maintenance agent of anesthesia during one-lung ventilation.

Moreover, Yondov et al. (7) investigated the halothane, isoflurane and propofol effects on pressure of arterial oxygen during OLV and also Schwarzkopf and colleagues (8) compared the effect of propofol as a maintenance agent with 1 MAC sevoflurane on patients during OLV and no significant differences in PaO2 was found between studied groups. Only in Kazuo Abe and his colleagues’ study (9), comparison of the effect of propofol, isoflurane and sevoflurane on arterial oxygenation during OLV showed an improved arterial oxygenation (greater arterial oxygen) with maintenance of propofol. This result is in contrast with our study and aforementioned studies that showed no statistical differences in PaO_2_ during OLV. Beck study (10) showed that propofol and sevoflurane similarly lead to a small increase in pulmonary shunt fraction. They also demonstrated that both drugs have the same hemodynamic effects on patients. Similarly, Kazuo Abe and his colleagues (9) observed the same effects of propofol, isoflurane and sevoflurane on patient’s` hemodynamics. In 2009, Schwarzkopf (8) showed the same hemodynamic parameters in propofol group and the group with inhaled anesthetic with 1 MAC sevoflurane. Since the results of the previous studies have supported our results, we can conclude that inhaled anesthetic agents (sevoflurane and isoflurane) produce the similar effects of propofol on hemodynamics (6). In contrast, Reid’s research (5) showed that mean heart rate was lower during TIVA than inhaled anesthetics but both groups had the equal effects on mean arterial pressure. In 2013, Modolo and his colleagues performed a systematic review including 20 studies with 850 participants that compared the intravenous anesthesia to inhaled anesthetics for one-lung ventilation. They found no evidence regarding the effect of maintenance anesthetic agents on participants’ outcomes (11). In addition, in another similar systematic review done by Bassi et al. (12), the same results were obtained. These results were in agreement with our results concerning the similar pressure of arterial oxygen, patients` blood pressure and heart rate in both groups.

Huang, in 2009, explained that propofol infusion may be more beneficial compared to isoflurane inhalation regarding oxidative stress (13). Another study in 2011 (14) which was comparing propofol with sevoflurane and desflurane, showed that volatile anesthetics were more effective in reducing the local alveolar inflammation but this effect was not systemic. In 2007, Schilling et al. investigated the effects of propofol and desflurane on alveolar inflammatory response during OLV. Based on their results, alveolar granulocytes percentage, IL-8, IL-10 and TNF were higher in propofol group (15). All these evidence confirmed the advantage of propofol in reduction of oxidative stress and the advantage of inhaled anesthetics in decreasing the local alveolar inflammation. These findings may support the role of underlying cause in diversity of these two drugs effects on gas exchange including pressure of arterial oxygen. In this study we did not follow the patients for long-term consequences and their survey and we also could not implement double blind approach due to structure of the intervention that took part in the same room with same personnel. We also could not use multiple observers for measurements, although as all measurements was done by digital equipment, it does not seems to make a big challenge in accuracy of the measurements. Based on our findings, using intravenous propofol or inhaled isoflurane as a maintenance anesthetic agent do not have different effect on pressure of arterial oxygen and patients` hemodynamics during two or one-lung ventilations.
